# Spatial Transcriptomics Identifies Expression Signatures Specific to Lacrimal Gland Adenoid Cystic Carcinoma Cells

**DOI:** 10.3390/cancers15123211

**Published:** 2023-06-16

**Authors:** Acadia H. M. Moeyersoms, Ryan A. Gallo, Michelle G. Zhang, Vasileios Stathias, Michelle M. Maeng, Dawn Owens, Rayan Abou Khzam, Yoseph Sayegh, Cynthia Maza, Sander R. Dubovy, David T. Tse, Daniel Pelaez

**Affiliations:** 1 Dr. Nasser Ibrahim Al-Rashid Orbital Vision Research Center, Bascom Palmer Eye Institute, Miller School of Medicine, University of Miami, Miami, FL 33136, USA; ahm107@miami.edu (A.H.M.M.);; 2 Sylvester Comprehensive Cancer Center, Miller School of Medicine, University of Miami, Miami, FL 33136, USA; 3 Department of Pharmacology, Miller School of Medicine, University of Miami, Miami, FL 33136, USA; 4 Department of Ophthalmology and Visual Science, Yale School of Medicine, New Haven, CT 06437, USA; 5 Dr. Kiran C. Patel College of Allopathic Medicine, Nova Southeastern University, Davie, FL 33314, USA; 6 Florida Lions Ocular Pathology Laboratory, Bascom Palmer Eye Institute, Miller School of Medicine, University of Miami, Miami, FL 33136, USA; 7 Department of Biomedical Engineering, University of Miami College of Engineering, University of Miami, Miami, FL 33136, USA

**Keywords:** lacrimal gland adenoid cystic carcinoma, spatial transcriptomics, rare cancer, transcriptomic signature

## Abstract

**Simple Summary:**

Adenoid cystic carcinoma of the lacrimal gland is a rare but aggressive cancer with poor long-term prognosis. Due to the rarity of this cancer, little is understood about its molecular makeup, hindering the development of targeted therapeutic options to manage the disease. In this study, we combine the power of bulk RNA sequencing and the resolution of spatial transcriptomics to uncover the transcriptomic landscape of the cancer and its surrounding microenvironment. We identified novel transcriptomic signatures for the various cellular compartments within these cancer specimens and identified a putative cancer stem cell cluster which had not previously been reported and may be related to treatment resistance responses. We uncover a specific transcriptomic signature attributable to cancer foci and distinguish it from differential signatures previously reported in these tumors which arise from stromal and other tumor microenvironmental compartments. Elucidation of a cancer specific signature can be potentially harnessed in the development of advanced treatment options.

**Abstract:**

Although primary tumors of the lacrimal gland are rare, adenoid cystic carcinoma (ACC) is the most common and lethal epithelial lacrimal gland malignancy. Traditional management of lacrimal gland adenoid cystic carcinoma (LGACC) involves the removal of the eye and surrounding socket contents, followed by chemoradiation. Even with this radical treatment, the 10-year survival rate for LGACC is 20% given the propensity for recurrence and metastasis. Due to the rarity of LGACC, its pathobiology is not well-understood, leading to difficulties in diagnosis, treatment, and effective management. Here, we integrate bulk RNA sequencing (RNA-seq) and spatial transcriptomics to identify a specific LGACC gene signature that can inform novel targeted therapies. Of the 3499 differentially expressed genes identified by bulk RNA-seq, the results of our spatial transcriptomic analysis reveal 15 upregulated and 12 downregulated genes that specifically arise from LGACC cells, whereas fibroblasts, reactive fibrotic tissue, and nervous and skeletal muscle account for the remaining bulk RNA-seq signature. In light of the analysis, we identified a transitional state cell or stem cell cluster. The results of the pathway analysis identified the upregulation of PI3K-Akt signaling, IL-17 signaling, and multiple other cancer pathways. This study provides insights into the molecular and cellular landscape of LGACC, which can inform new, targeted therapies to improve patient outcomes.

## 1. Introduction

Lacrimal gland adenoid cystic carcinoma (LGACC) is a rare orbital malignancy notorious for its unpredictability and universally devastating lethality. Despite various permutations of the bimodal locoregional approach of surgery and radiation therapy, the survival rate is less than 50% at five years and less than 20% at ten years [1]. A recent long-term follow-up study reported improved survival outcomes by integrating neoadjuvant intra-arterial cytoreductive chemotherapy (IACC) as the third arm of a trimodal strategy [2]. Despite the salutary advantage of an IACC-anchored trimodal protocol in achieving local disease control, disease relapse may occur 5–15 years later. To augment the shortcomings of conventional chemotherapy, surgery, and radiation therapy treatment options, a more thorough understanding of the molecular makeup of LGACC is required to identify new and effective targeted treatments [3,4,5].

Malignant transformations in LGACC, as with other ACCs, occur due to amplifications and translocations of the MYB proto-oncogene. Subsequent activating mutations in the NOTCH pathway correlate with more aggressive tumor phenotypes and poor prognosis [6]. Unfortunately, MYB and NOTCH are currently not targetable molecules, and efforts to indirectly inhibit them have not been successful in stopping ACC progression. Thus, exploring the transcriptomic signatures elicited by these genomic aberrations remains the most fruitful space in discovering LGACC-specific therapeutic interventions.

Initial identification of tumor-relevant pathobiological signatures is routinely performed by analyzing differential expression profiles between malignant and healthy control tissues. When performed on bulk tissue specimens, however, this approach fails to discern tumor-specific signatures from those of surrounding and ‘contaminating’ phenotypes, which can obscure the underlying tumor biology. This is especially true for tumors that are typically excised en bloc with other tissue structures and for which obtaining primary untreated specimens is not feasible as is the case for LGACC. In this study, we combine bulk RNA sequencing (RNA-seq) and spatial transcriptomics to investigate the key genes and pathways specifically enriched in LGACC clusters within excision specimens. This approach permits a high-resolution interrogation of LGACC in which we distinguish microenvironment and stromal components from tumor cell signatures. Through our analyses, we identify potential tumor-specific biomarkers and targetable pathways. These insights may facilitate the development of ACC-specific targeted therapies that can enable globe-sparing approaches to LGACC management. We also demonstrate that some of the most significant differential changes reported in the ACC literature by bulk RNA-seq do not arise from tumor cells but originate from normal surrounding tissues, skewing their value as potential therapeutic targets.

## 2. Materials and Methods

### 2.1. Bulk RNA-Seq and Analysis

LGACC samples (*n* = 5) were post-IACC treatment, excised specimens. Normal lacrimal gland samples were from non-LGACC patients. Samples were immediately flash-frozen. Tissue samples were subjected to RNA extraction using a Direct-zol RNA kit (Zymo Research, Irvine, CA, USA). Purified total RNA from 7 samples, 5 LGACC and 2 normal lacrimal gland controls (noncancerous), were sent for library preparation and sequencing (Onco-Genomics Shared Resource, University of Miami Miller School of Medicine, Miami, FL, USA). RNA quality was based on an RNA integrity number (RIN) score with the minimum threshold set at 6 to proceed with sequencing. Paired-end sequencing with 30–40 million reads was performed on a NovaSeq 6000 (Illumina, San Diego, CA, USA). After assessing the raw fastq files for quality with FastQC, reads were trimmed with trimadapt and aligned against the human genome (GRCh38) using STAR. Gene counts were identified as counts per million (CPM) from STAR output and imported into R Studio for differential gene expression (DGE) analysis using edgeR (Version 3.40.1). Genes with a minimum CPM of 5 were removed, and the remaining genes were normalized. Stringent filtering analysis was carried out for DGE analysis using an FDR value < 0.05 and logFC > 2 or <−1. See the data availability section for gene expression data.

### 2.2. Spatial Transcriptomics

Spatial transcriptomics was completed using a 10× Genomics Visium formalin-fixed paraffin-embedded (FFPE) Spatial Transcriptomics kit (10× Genomics, Pleasanton, CA, USA). Spatial transcriptomics was conducted on one post-IACC archival FFPE LGACC sample classified at T4bN0M0 in accordance with AJCC TNM Classification 7th Edition that was from a different patient from bulk RNA-seq. RNA was extracted from 3 FFPE 10 µm slivers per sample using a Qiagen RNeasy FFPE Kit (Qiagen, Hilden, Germany), and DV200 quality was measured using the 2100 BioAnalyzer system (Agilent Technologies Inc., Santa Clara, CA, USA). A sample section was selected by pathology that encompassed the cancer and surrounding tissue, and the quality was deemed acceptable by collaboration with 10× Genomics to proceed with the experiment.

The region of interest was selected by pathology that included the cancer and surrounding noncancerous tissue. FFPE block was cut at 10 µm thickness and mounted on a 10× Visium (FFPE protocol, version 1) (10× Genomics, Pleasanton, CA, USA) slide according to the manufacturer’s specifications. The slide was dried in an oven for 2 h, followed by drying overnight at room temperature in a desiccator. The following day, the slide was heated on a thermocycler adapter for 2 h at 60 °C. Hematoxylin and eosin (H&E) staining was performed according to standard procedures. A temporary coverslip was applied using 80% glycerol for imaging and then easy removal in water. Images of H&E stained slides were captured using Leica Aperio AT2 (Leica Microsystems, Exton, PA, USA). Sections were permeabilized, and cDNA synthesis was performed on-slide. The cDNA generated was used for library preparation according to the manufacturer’s instructions. Paired-end sequencing was performed using NovaSeq 6000 (Illumina, San Diego, CA, USA) by the Onco-Genomics Shared Resource (University of Miami Miller School of Medicine, Miami, FL, USA, 33137).

### 2.3. Pathology Annotations

The H&E image of the sample was read and annotated by an experienced ophthalmic pathologist (Figure S2). The H&E stained slide and the sequencing results were analyzed using cluster analysis. The annotated classifications were compared with the computational clustering analysis of the transcriptome for similarity.

### 2.4. Spatial Transcriptomic Analysis

SpaceRanger pipeline was used to process and align FASTQ files to the human reference genome GRCh38. Following SpaceRanger processing, counts were processed in Seurat R Program (Version 4.3.0) [7,8,9,10]. Cells with no expression were removed. Counts were normalized using the SCTransform command that accounts for biological variance and visualized with SpatialFeaturePlot (Figure S3A). We performed principal component analysis (PCA) and Seurat’s FindClusters function to determine the ideal resolution (0.8) for discerning discrete clusters in the cluster analysis (Figure S3B). Cluster identification and differential gene expression analysis were performed using the Seurat commands RUNPCA, FindNeighbors, FindClusters, and RUNUMAP. All spatial transcriptomic figures were made using Seurat commands. Monocle3 (Version 1.3.1) was used to perform trajectory analysis [11,12,13]. See the data availability section for cluster gene expression data.

### 2.5. Gene Ontology (GO) and Pathway Analyses

Overexpressed and downregulated genes in each cluster were investigated via gene ontology and pathway analysis. RNA-seq differentially expressed genes were analyzed for downstream GO and pathway analysis using Database for Annotation, Visualization, and Integrated Discovery (DAVID) v6.8 Functional Analysis Tool using GOTERM DIRECT ontologies and KEGG pathway analysis on differentially expressed genes [14]. GO and pathway plots were generated with ggplot2 (Version 3.4.0) [15].

### 2.6. Immunofluorescent Staining

Post-IACC archival LGACC sections were cut at 10 µm thickness (*n* = 3). Slides were incubated in primary antibodies diluted in PBS at 4 °C overnight (Table S1). Slides were then incubated with AlexaFluor (Abcam, Cambridge, UK) secondary antibodies (1:200) for 2 h. SlowFade Diamond Antifade Mountant with DAPI (Thermo Fisher Scientific, Waltham, MA, USA) was used. Staining was conducted on the entire tissue slide. Imaging was performed on a Leica AOBS SP8 confocal microscope (Leica Microsystems, Exton, PA, USA).

## 3. Results

### 3.1. Bulk RNA-Seq

**Figure 1:**
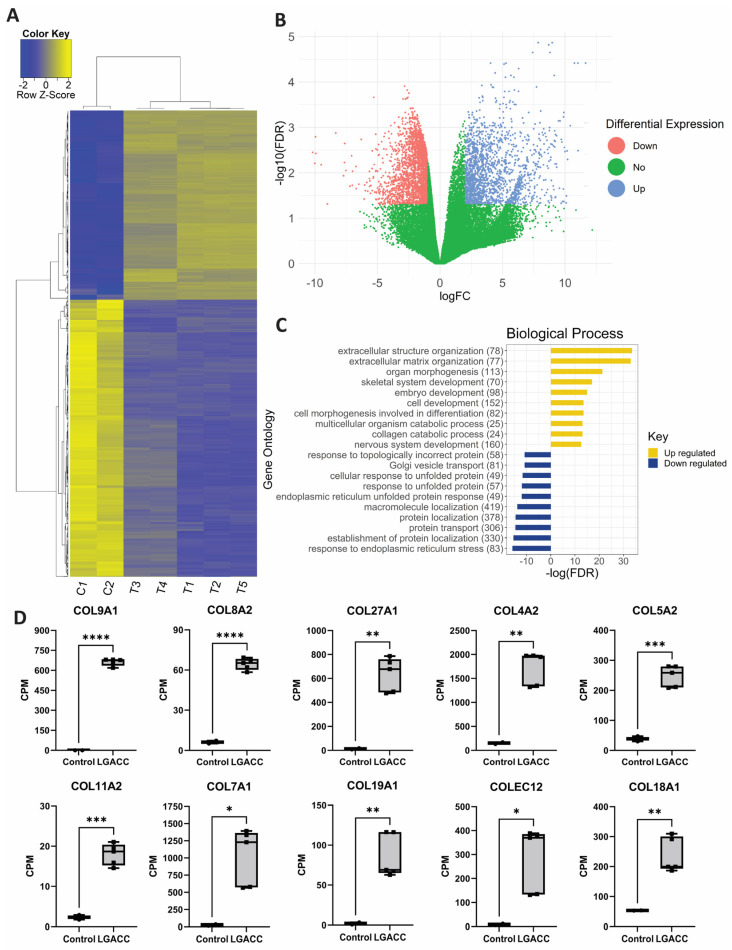
RNA sequencing analysis of LGACC vs. normal lacrimal gland. (**A**) Heat map of top 1000 differentially expressed genes of normal control (C) samples (*n* = 2) vs. LGACC (T) samples (*n* = 5). (**B**) Volcano plot of logFC by −log10(FDR) showing differentially expressed genes with logFC cutoff > 2 and <−1 and FDR < 0.05. Red and blue dots represent downregulated and upregulated genes, respectively. (**C**) Biological process gene ontology analysis of up- and downregulated genes. *X*-axis represents −log10 (*p*-value) of gene ontology value. Downregulated gene ontologies are represented with a negative value. (**D**) Box plots of RNA-seq counts per million (CPM) values for the top 10 differentially expressed collagens comparing normal lacrimal gland with LGACC. Statistical significance indicated by astricks represented as follows: * *p* value less than 0.03, ** *p* value < 0.01, *** *p* value less than 0.001, and **** *p* value < 0.0001.

The results of our bulk RNA-seq analysis identified 3499 differentially expressed genes (DEGs) between LGACC (*n* = 5) and normal lacrimal gland from healthy donors that have been identified to not have any related disease prior to sample acquisition (*n* = 2). Of the DEGs, 1228 and 2271 were upregulated and downregulated, respectively, in LGACC compared with normal lacrimal gland. The top 1000 most significant genes evaluated by the lowest FDR value are represented in a heat map (Figure 1A). Filtering for significant DEGs was carried out with stringent criteria to discern genes that differentiate LGACC to normal lacrimal gland (Figure 1B). The results of the two-dimensional principal component analysis (PCA) showed distinct clustering of the control and cancer samples, which separately clustered into two groups, potentially due to the sample size (Figure S1A). The results of the gene ontology (GO) analysis revealed that the extracellular matrix (ECM) is the leading upregulated biological process, cellular component, and molecular function (Figure 1C and Figure S1B,C). The most downregulated GO terms included the immune response for biological processes, the integral component of the membrane for cellular components, and the metalloendopeptidase activity for molecular function.

Since the ECM was the most upregulated GO term, we specifically looked at different collagen gene expression levels. We plotted the top 10 differentially expressed collagens from RNA-seq to show the consistent, high overexpression of collagens in our cancer samples (Figure 1D). The collagen CPM for the normal samples is nonzero, indicating the expression detection of collagen transcripts, albeit significantly lower than that for the LGACC samples.

### 3.2. Spatial Transcriptomics

**Figure 2:**
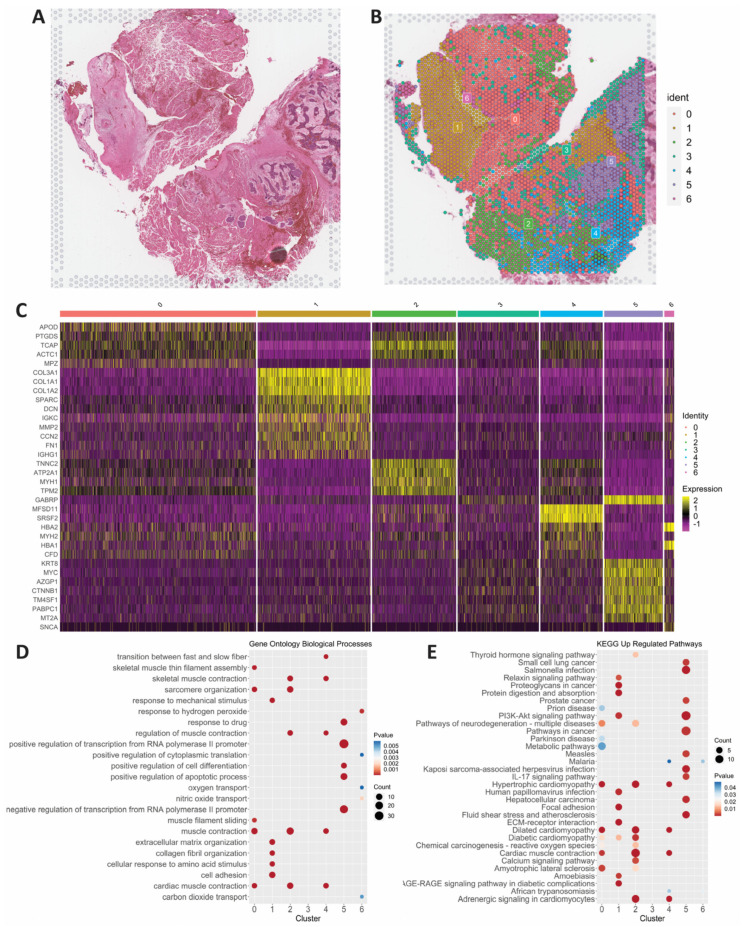
Spatial transcriptomics elucidates LGACC and surrounding tissue signatures. (**A**) Hematoxylin–eosin (H&E) staining of LGACC section. (**B**) Spatial transcriptomic clustering results overlaid on H&E stained section. (**C**) Heat map of top markers for each cluster based on PCA. (**D**) Gene ontology biological process analysis of upregulated genes for each cluster. (**E**) KEGG pathway analysis of upregulated genes for each cluster.

**Figure 3:**
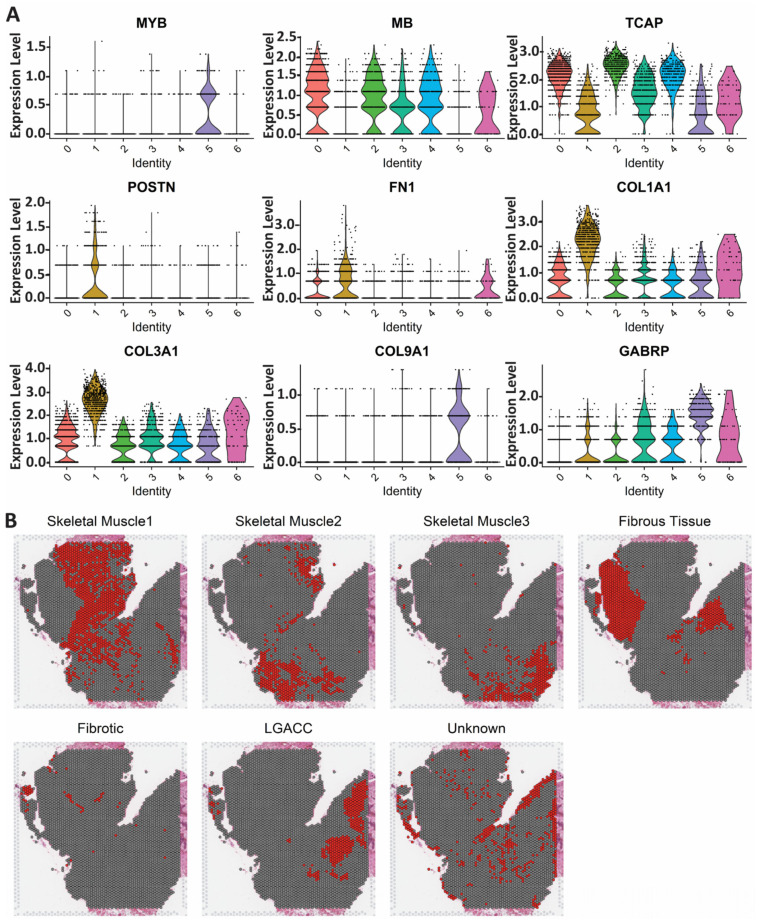
Determining the identity of each cell cluster in spatial transcriptomic sample. (**A**) Violin plots of genes specific to cell type and top expressing genes in clusters. (**B**) Spatial plots showing the location of each cluster named by cell type determined from analysis.

We performed spatial transcriptomics on an LGACC that had undergone IACC treatment (Figure 2A). About 97.1% of reads to the probe set were mapped with 16,624 genes detected. The results of our analysis identified 7 cell clusters (Figure 2B) and identified the top expressing genes through finding all markers for each cluster and plotting the top 10 genes for each (Figure 2C). Based on the top expressed genes, differential gene expression, pathology annotations, biological processes, and pathway analysis of each cluster, we identified phenotypes associated with each cluster (Figure 2C–E). Based on gene expression and histology, clusters 0, 2, and 4 are skeletal muscle due to the high expression of APOD, MB, TCAP, and TNNC2 with significant upregulation of contractile components and cardiomyopathy processes (Figure 2C–E and Figure 3A,B). Cluster 1 is fibrous tissue based on its high expression of multiple collagens, DCN, and SPARC, as well as the upregulation of cell adhesion, extracellular matrix, and other reactive processes (Figure 2C–E and Figure 3A,B). Cluster 6 only had 8 genes overexpressed when compared with the other clusters, including HBA1 and 2, SNCA, and EEF2. The top upregulated biological process is the response to hydrogen peroxide (Figure 2C–E). Based on the distinct transcriptomic signature and the pathologist’s annotations, we identified cluster 6 as reactive fibrotic cells (Figure 3B).

Cluster 5 was the only cell cluster with an expression of MYB, the most common gene upregulation signature in LGACC tumors, and a high expression of GABRP, MYC, and other proto-oncogenes (Figure 2C and Figure 3A). Upon the downstream analysis of processes and pathways, cluster 5 had multiple cancer signatures including small-cell lung cancer, PI3K-Akt signaling, and pathways in cancer significantly upregulated. Pathology annotations also confirmed that cluster 5 encompasses the malignant LGACC phenotype (Figure 2D,E and Figure S2).

**Figure 4:**
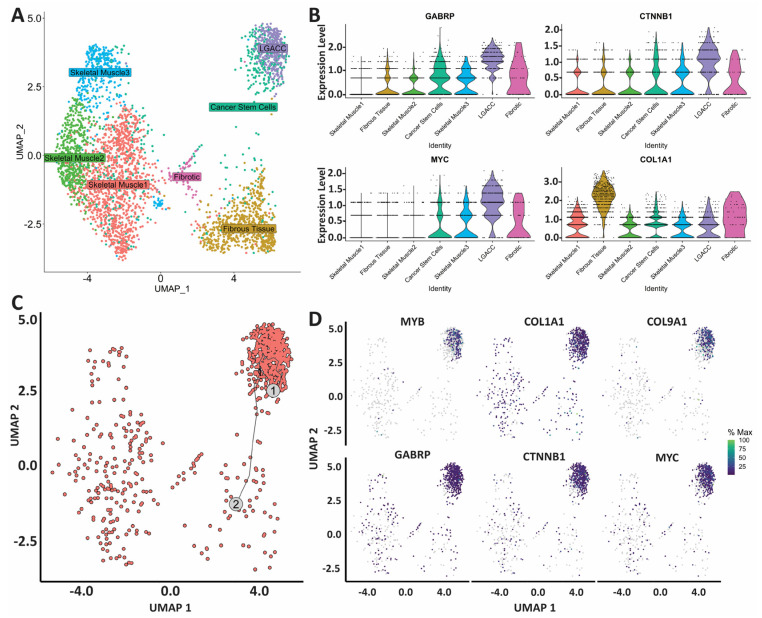
Characterization of cluster 3 as potential transition or cancer stem cell cluster. (**A**) UMAP of cell cluster identities. (**B**) Violin plots of gene signature of cluster 3 alone and when compared with muscle cell clusters. (**C**) Monocle trajectory map indicating a zoomed-in UMAP of cluster 5 (LGACC) (indicated as 1 on the graph) as the primary and cluster 3 (indicated as 2 on the graph) cells coming from the malignant cluster. (**D**) UMAPs of genes of interest for cluster 3 found in trajectory analysis.

Cluster 3 presents transcriptionally distinct from the LGACC cluster (cluster 5) and from the surrounding supportive tissue, but histologically, it appears to be both integrated among the cancer area, as well as abutting the cancer section (Figure 4A and Figure S2). With spatial transcriptomics, we have identified a novel cancer stem cell cluster that abuts the LGACC tumor foci that is unique at the transcriptomic level. We performed Monocle trajectory analysis to investigate whether cluster 3 may be a precursor to cluster 5 or if it may arise from cluster 5. Trajectory analysis utilizes statistical models to identify the cell state order and can predict the lineages of cells [11]. Based on this analysis, cluster 3 derives from cluster 5 (Figure 4C). We individually compared cluster 3 with clusters 0 through 5 to identify the pathways upregulated in this cluster (Figure S4). Cluster 3 was found to have more of a muscle-like phenotype when compared with fibrous tissue (cluster 1) and LGACC (cluster 5) (Figure S4B,E). Yet when compared with the muscle cell clusters (clusters 0, 2, and 4), cluster 3 differential gene signatures were enriched for cancer-specific pathways (Figure S4A,C,D). Pointedly, when compared with muscle cell clusters, cluster 3 significantly overexpresses GABRP and MYC (also differentially overexpressed in the LGACC cluster), as well as CTNNB1 and COL1A1 (Figure 4B). We visualized the gene expression patterns of MYB, COL1A1, COL9A1, GABRP, CTNNB1, and MYC in the Monocle trajectory (Figure 4D).

### 3.3. Combination of Bulk RNA-Seq and Spatial Transcriptomics Identifies Markers of LGACC and Surrounding Tissue

**Figure 5:**
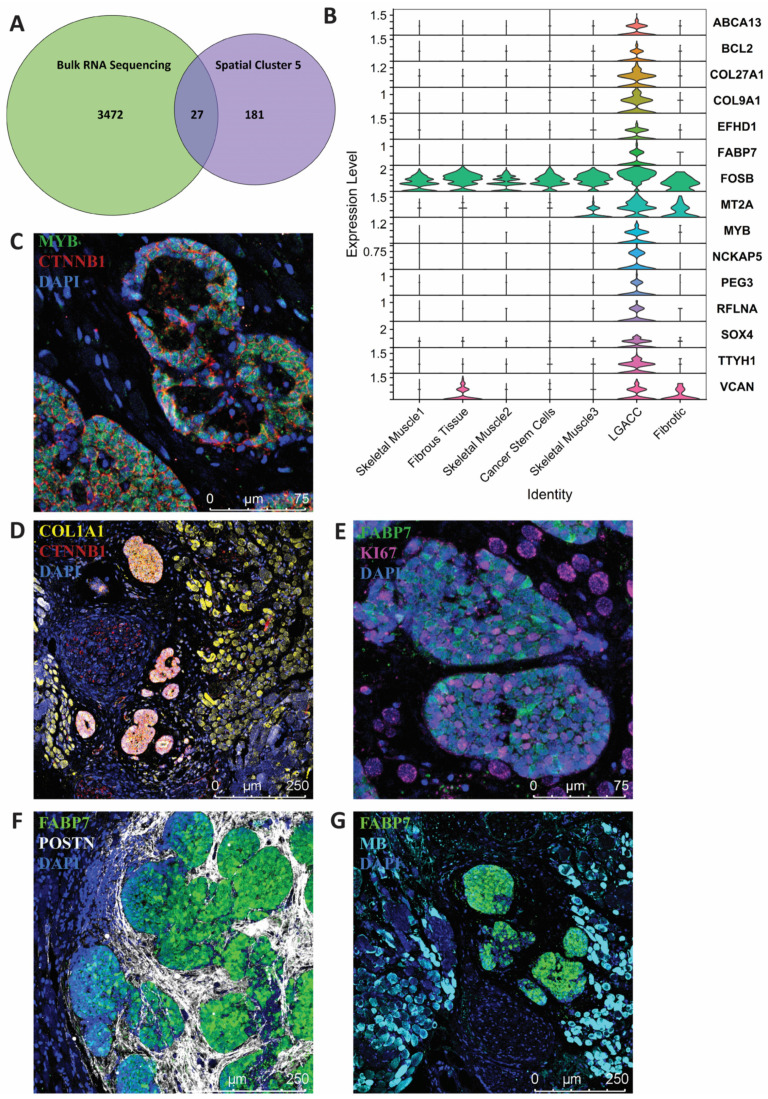
LGACC-specific signature identified through spatial transcriptomics and bulk RNA sequencing. (**A**) Venn diagram representing differentially expressed genes from bulk RNA sequencing and spatial transcriptomics and the overlap found between the two. (**B**) Stacked violin plot of 15 overexpressed genes that are found in both bulk and spatial transcriptomics. (**C**–**G**) Immunofluorescent staining of LGACC-specific signature and surrounding tissue signatures.

In our spatial transcriptomic analysis, we were able to discern a gene expression signature restricted to LGACC tumor foci. We then overlaid our bulk RNA-seq analysis to the spatial LGACC cluster (cluster 5) to elucidate an LGACC-specific transcriptomic signature. Spatial transcriptomics is extremely beneficial in discerning expression signatures restricted to specific cell types within a sample but lacks the sequencing depth of other NGS platforms. The results of our integrated analysis identified 27 statistically significant genes that are differentially expressed in the RNA-seq data and have the same directional expression in spatial transcriptomics (Figure 5A).

We also confirmed the expression and LGACC-specific localization of several markers found in our LGACC signature through immunofluorescent staining. Immunofluorescent staining selection was determined by expression level, antibody availability, and antibody performance. We conducted staining for fatty acid binding protein (FABP7) (Figure 5E–G), MYB (Figure 5C and Figure S5), and β-catenin (CTNNB1) (Figure 5C,D and Figure S5). In contrast, periostin (POSTN), which is significantly upregulated in bulk RNA-seq but was not included in our integrated bulk-spatial transcriptomic analysis, was demonstrated by immunofluorescent staining to be a marker in fibrous tissues surrounding cancer clusters (Figure 3A and Figure 5E). Collagen type 1 alpha chain 1 (COL1A1) is not specific to just LGACC; it is also expressed in the surrounding fibrotic and muscle tissue (Figure 3A and Figure 5D). We used myoglobin (MB) as a muscle marker that was noted in the spatial transcriptomic analysis to stain the surrounding muscle compared with the cancer in the immunofluorescent images (Figure 5F). All evaluated immunofluorescent staining of LGACC samples exposed specific signatures that further confirmed our bulk RNA-seq results (Figure 5 and Figure S5).

## 4. Discussion

LGACC is a highly aggressive and lethal cancer for which effective treatment remains elusive. Understanding the molecular characteristics specific to LGACC is critical for further treatment development, molecular monitoring of disease progression, and overall enhanced management. Through integrated bulk RNA-seq and spatial transcriptomics in this study, we elucidate the LGACC-specific component within the landscape of differential expression signatures between malignant ACC and normal excretory gland tissues. Even with the rarity of this cancer, we were able conduct bulk RNA sequencing on 5 tumor samples and further validate and characterize this signature with an additional sample for spatial transcriptomics allowing for an in-depth transcriptomic analysis. From the bulk RNA signature, we identified the signature of LGACC and the surrounding supportive tissue using spatial transcriptomics. We deconvoluted the gene expression signature from the muscle and fibrous tissue that we had observed in the bulk RNA-seq and were able to validate the LGACC-specific signature from the bulk sequencing with spatial transcriptomics. This signature can inform the development of novel targeted treatments for ACC while reducing setbacks of targeting non-ACC differential markers present in these types of specimens.

The current standard of practice in the management of LGACC uses IACC or proton therapy prior to the removal of the tumor. Due to this treatment schedule, it is extremely rare to obtain primary untreated samples of this tumor; thus, our study is performed on post-IACC samples, and the results presented must be contextualized as such [4]. Due to this limitation, and because spatial transcriptomics provides insights about a singular point in time of the tumor state, we cannot discern a precise evolution for any cell state in our specimens beyond that which is produced by lineage prediction algorithms currently available.

One particular cluster of interest (cluster 3) was not readily identifiable solely based on its transcriptional and/or pathological features. Physically, cluster 3 surrounds and abuts LGACC foci. On UMAP projections, its transcriptomic signature approximates it to the LGACC cluster. To further elucidate the biological relevance of cluster 3, we performed Monocle trajectory analysis to determine if this cluster is a precursor transition state or cancer stem cells arising from the malignant cluster. The results of our analysis suggest that cluster 3 results from the LGACC cluster, leading us to hypothesize that it is a cancer stem cell (or otherwise quiescent ACC) cluster, presumably arising from post-IACC treatment reactivity. Further evidence for our hypothesis was determined with the indication of the following cancer stem cell markers: GABRP, CTNNB1, and MYC, which are seen in breast, colon, and lung cancer stem cells [16,17,18]. From the available data and knowledge, we advance this hypothesis of a cancer stem cell or quiescent LGACC phenotype, which has never been characterized in LGACC. Cancer stem cells are a subgroup of tumor cells that can arise and further drive cancer progression and metastasis. They can arise from response to treatments and hold different genetic mutations that allow them to be more resistant to treatment modalities [19,20,21]. Cancer stem cells play an important role in treatment resistance, tumor recurrence, and aggression of cancer. Identifying cancer stem cell populations in a tumor can give insight to potential drivers of recurrence and metastasis, which is common in LGACC. Interestingly, cluster 3 has a unique signature that distinguishes it from normal muscle as well as from the well-defined cancer cell cluster. Compared with the muscle cell clusters, this cluster has an upregulation of CTNNB1, GABRP, and MYC. CTNNB1 is part of the Wnt/β-catenin pathway, which is commonly known to have mutations in various cancers in which it leads to advanced disease and is difficult to target. The upregulation of this pathway has been shown in cancer stem cells that drive metastasis [22,23,24,25,26]. The overexpression of GABRP has been shown to maintain stem cell characteristics in breast cancer, providing support in chemoresistance and promoting migration [16,27]. MYC overexpression has been noted in addition to CTNNB1 in cancer stem cells, as it is a transcription factor that controls cell growth, differentiation, and cell maintenance. MYC’s role in cancer stem cells is unique as it can perpetuate stemness and drive malignancy, both important phenotypes in cancer stem cells. MYC is also a key driver in cancer stem cells of breast and colorectal cancers [17,28,29,30].

We performed gene ontology analysis of cluster 3 compared with the LGACC cluster and noted the upregulation of epithelial to mesenchymal transition (EMT). This could be a signature indicative of this cluster becoming more stem cell in nature. It has been shown that EMT drives cancer progression, leads to more stemness, drives metastasis, and results in chemoresistance [27]. When compared with the muscle cell clusters, ECM pathways and ontologies are noted to be increased. The ECM plays a crucial role in the tumor microenvironment in many cancers, and it was the most upregulated biological process in our bulk RNA-seq. The ECM is critically important for cancer progression, pivotal in the migration and death evasion processes [31,32]. Arolt et al. examined the role of the ECM in salivary gland carcinomas, identifying how it is essential for cancer progression and can be involved in chemoresistance development [33]. Other studies have demonstrated a connection between ECM overexpression and invasion [34,35,36]. The cancer stem cells could be supported by the upregulation of ECM properties or could be driving the overexpression itself, thus promoting cancer progression. LGACC has a poor prognosis due to early and pervasive perineural invasion in which the involvement of collagens and the ECM could be potential mediators.

Top overlapping differentially expressed genes from our integrated analysis of bulk RNA sequencing and spatial transcriptomics have essential roles in cancer such as cell proliferation and motility. In the overlap analysis, we found the overexpression of ABCA13, PEG3, and SOX4 that have been shown to be poor prognostic markers in other cancers including ovarian, glioblastoma, and colon [37,38,39,40]. We observed a significant increase in FABP7 levels as measured by RNA-seq and spatial transcriptomics and validated in immunofluorescence. In the spatial transcriptomic analysis, this transcript was explicitly overexpressed in the LGACC cluster. This validates our findings that it is a LGACC-specific marker and not expressed in surrounding supportive tissues. FABP7 is a cytoplasmic protein involved in gene regulation and lipid metabolism. It is upregulated in a wide range of diseases that include breast cancer, melanoma, and optic nerve glioma [41,42]. FABP7 is crucial for essential cell functions such as proliferation and signaling [43]. Its role has been described in a wide range of cancers as being upregulated. FABP7, as well as COL27A1, has been seen to be upregulated in head and neck adenoid cystic carcinoma [44]. When specifically targeted with shRNA knockdowns, cancer cell proliferation decreases [41,45,46,47]. The overexpression of FABP7 in LGACC can be a key driver in the proliferation of cancerous cells, leading to its potential as a candidate molecule to specifically target LGACC.

Brayer et al. conducted RNA-seq on salivary, breast, lacrimal gland, and cutaneous ACC samples. We contributed to this study by providing LGACC samples (*n* = 6). Upon gene expression analysis comparing the different ACC subtypes, COL27A1 and FOSB were identified as the top overexpressed genes for LGACC in the Brayer paper, validating our findings in our overlap analysis. Additionally, ABCA13, EFHD1, and FOSB were noted as poor outcome predictive markers for ACCs, and we had seen these genes to be overexpressed in our comparison analysis [48].

Identifying a cancer-specific signature provides the opportunity to identify potential targeted therapies that can enhance the management of LGACC. While known driver mutations in MYB and the activation of Notch are not actionable targets right now, we have identified the BCL2 gene overexpression solely in LGACC cancer cells that has small molecular inhibitors including Gossypol and Disarib [49,50,51,52,53]. This discovery can offer a potential targeted treatment for this disease.

From our bulk RNA-seq and spatial transcriptomics overlap analysis, we elucidated specific molecular characteristics of LGACC. However, spatial transcriptomics and bulk RNA-seq only give insight into a static state of the tissue and do not give insight into the origination and progression of the disease. We cannot discern if cluster 3 developed before or after IACC treatment and cannot ascertain its role in cancer progression. Further studies of single-cell RNA-seq before and after treatment can provide more insight into the development of the heterogeneity of the tumor, provided that such samples become available for study.

## 5. Conclusions

Current treatments for LGACC are limited and radical, typically including exenteration of the orbital socket followed by high-dose chemoradiation. Despite this harsh approach, recurrence, metastasis, and disease-specific mortality rates remain extremely high. Our identification of the LGACC-specific transcriptome allows us to study the molecular characteristics of LGACC in isolation of the confounding noise in this specific cancer environment. Furthermore, this analysis provides the molecular foundation to design targeted therapeutic interventions that can enhance patient outcomes and LGACC clinical management.

## Data Availability

Bulk RNA-seq and spatial transcriptomics expression data for all samples are available in Gene Expression Omnibus (GEO). GEO accession numbers: bulk RNA-seq (GSE228914) and spatial transcriptomics (GSE228685).

## Supplementary Materials

The following supporting information can be downloaded at: https://www.mdpi.com/article/10.3390/cancers15123211/s1, Figure S1: LGACC and normal sample comparisons via RNA sequencing downstream analysis; Figure S2: Pathology annotated spatial transcriptomic sample section; Figure S3: Spatial transcriptomic gene counts and cluster resolution determination; Figure S4: Pathway analysis for cluster 3 individually compared with other clusters to characterize signature; Figure S5: Immunofluorescent antibody validation; Table S1: Antibodies utilized for immunofluorescent staining.
