# Supplementary material for: Spatial Transcriptomics Identifies Expression Signatures Specific to Lacrimal Gland Adenoid Cystic Carcinoma Cells
Source: Cancers (Basel). 2023 Jun 16;15(12):3211. doi: 10.3390/cancers15123211 (PMC10296284; doi:10.3390/cancers15123211)
Supplement: Supplementary file 1 [file cancers-15-03211-s001.zip › cancers-2440348-supplementary.pdf]

Supplementary Materials: Spatial Transcriptomics Identifies Expression Signatures Specific to Lacrimal Gland Adenoid Cystic Carcinoma Cells

Acadia H. M. Moeyersoms, Ryan A. Gallo, Michelle G. Zhang, Vasileios Stathias, Michelle M. Maeng, Dawn Owens, Rayan Abou Khzam, Yoseph Sayegh, Cynthia Maza, Sander R. Dubovy, David T. Tse and Daniel Pelaez

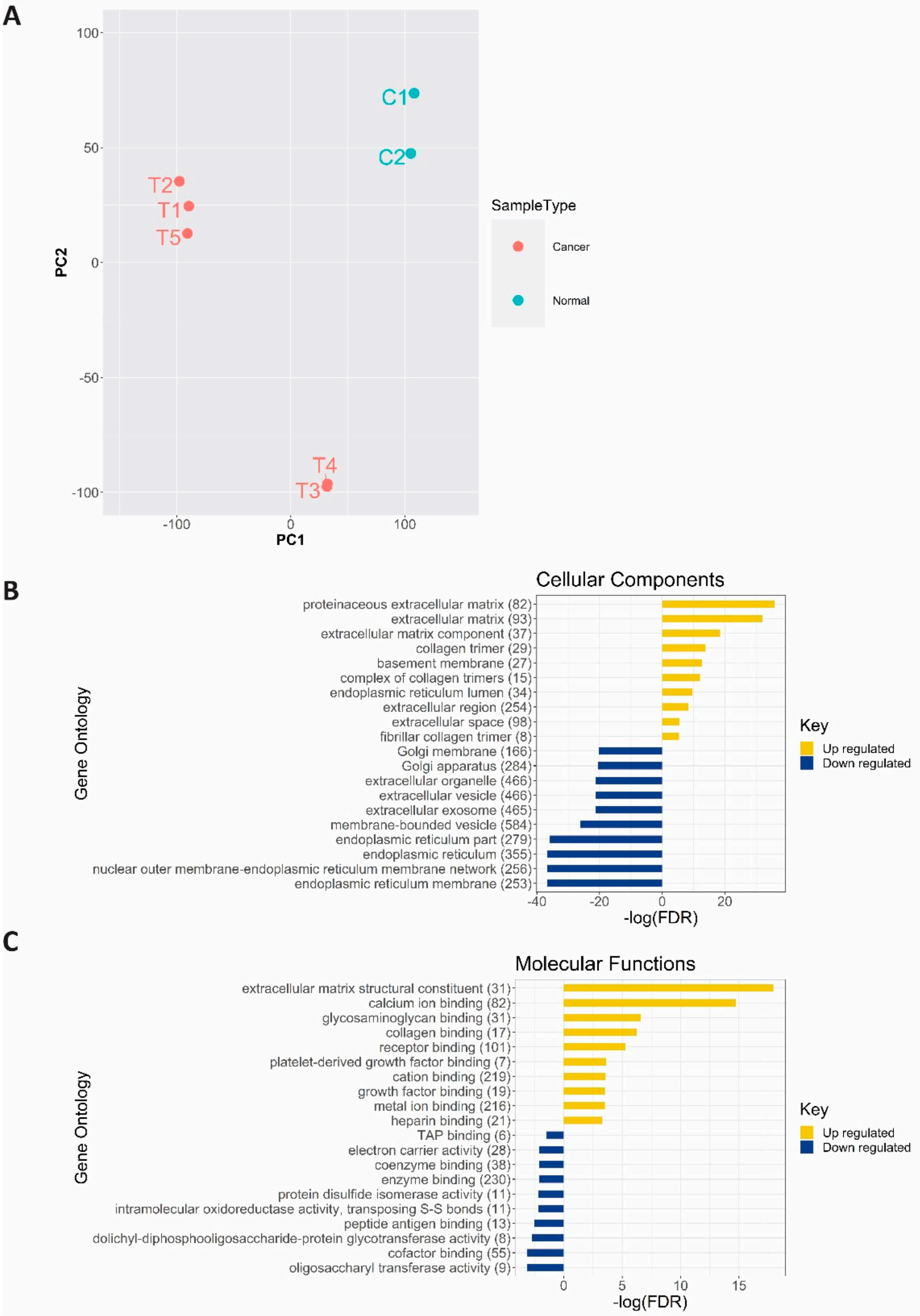

Figure S1. LGACC and normal sample comparisons via RNA sequencing downstream analysis. **(A)** PCA plot of 7 RNA sequencing samples. Red and teal represent cancer and normal samples,

respectively. **(B)** Cellular component gene ontology analysis of up and down-regulated genes. **(C)** Molecular function gene ontology analysis of up and down-regulated genes. For both B and C, x-axis represents -log10(p-value) of gene ontology value with the down regulated ontologies represented with a negative value.

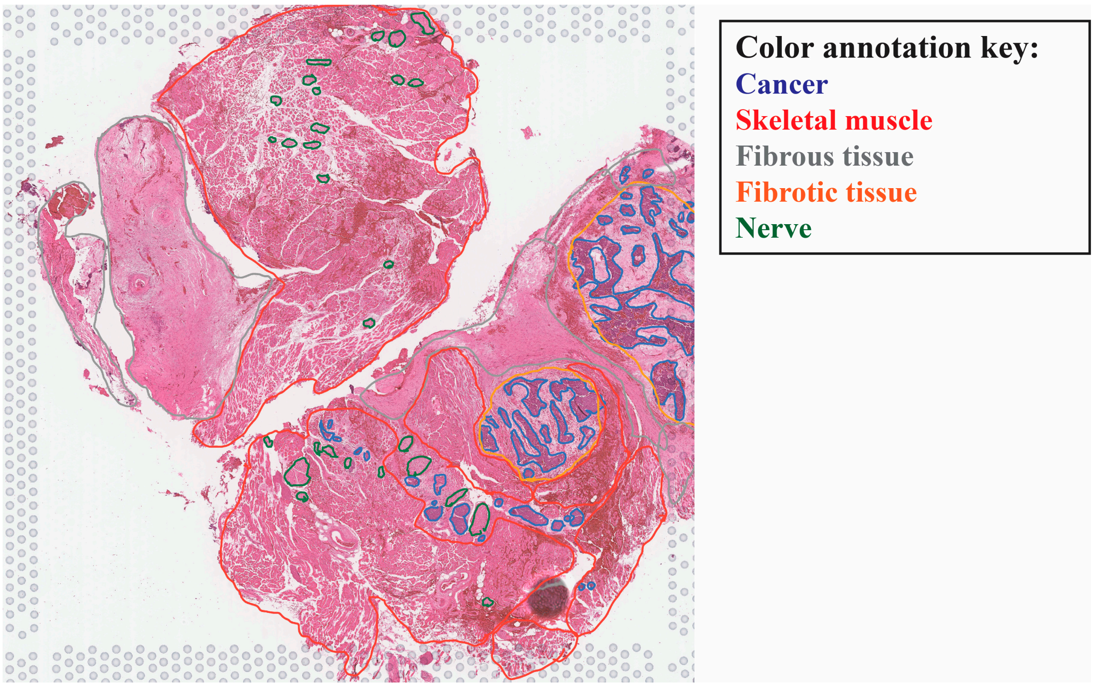

Figure S2. Pathology annotated spatial transcriptomic sample section. Pathologist annotated the H&E stained section of LGACC, fibrous tissue, fibrotic, skeletal muscle, and nerve. The figure key identifies which color corresponds to which tissue type.

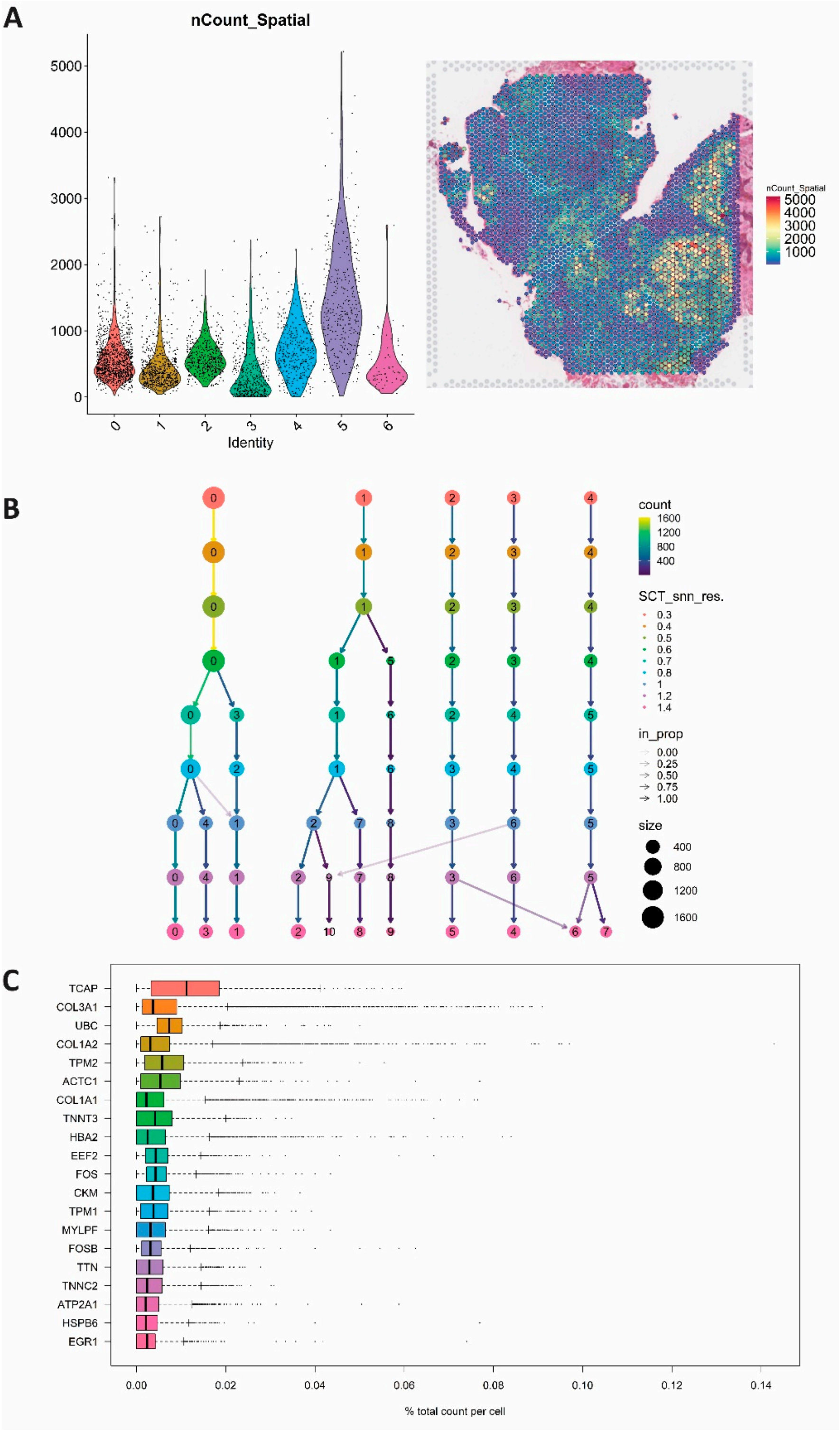

Figure S3. Spatial transcriptomic gene counts and cluster resolution determination. **(A)** Violin plot for total number of counts per cluster and spatial total counts over entire sample. **(B)** Hierarchical cluster tree to determine ideal cluster resolution to discern discrete cell types. **(C)** Box and whisker plot of highest expressed genes throughout spatial transcriptomic sample.

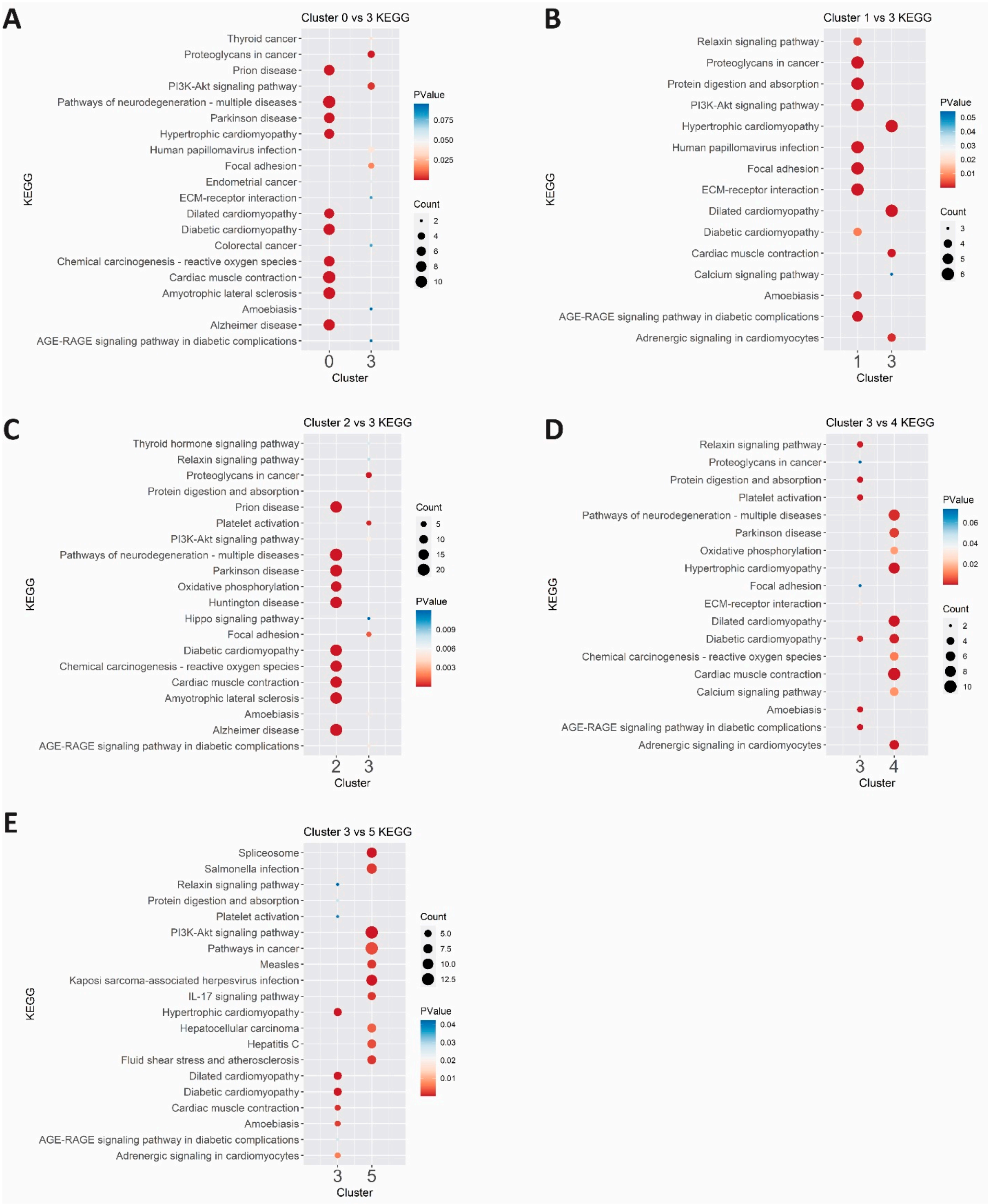

Figure S4. Pathway analysis for cluster 3 individually compared to other clusters to characterize signature. **(A, B, C, D, E)** Cluster 3 compared to clusters 0, 1, 2, 4, and 5 respectively, and the top 10 pathways upregulated in cluster 3 and the cluster it is compared to.

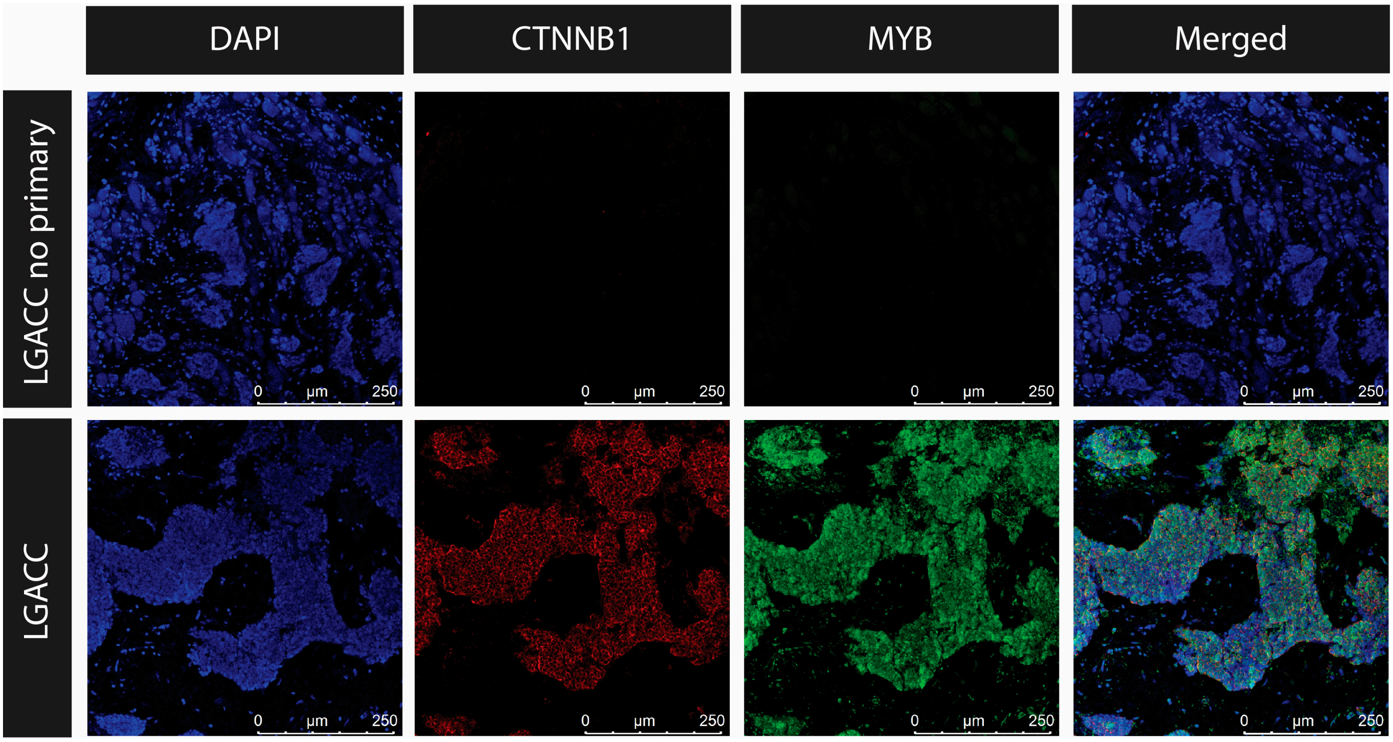

Figure S5. Immunofluorescent antibody validation. Top row panels represent LGACC sample with no primary antibody as a negative control for secondary antibody autofluorescence. Bottom row panels represent LGACC sample with primary antibody staining for CTNNB1 and MYB.

Table S1. Antibodies utilized for immunofluorescent staining.

Target	Company	Catalog Number	Dilution
MYB	Bethyl Laboratories, Montgomery, Texas, USA	A304-135A	1:400
FABP7	Abcam, Cambridge, UK	ab279649	1:200
KI67	Novus Biologicals, Englewood, CO, USA	NBP2-22122	1:100
GABRP	Thermo Fisher Scientific, Waltham, MA, USA	BS-13257R	1:50
CTNNB1	Abcam, Cambridge, UK	ab19381	1:100
COL1A1	Thermo Fisher Scientific, Waltham, MA, USA	PA5-29569	1:100
MB	Thermo Fisher Scientific Waltham, MA, USA	MA5-33110	1:100
POSTN	Thermo Fisher Scientific Waltham, MA, USA	A500070	1:100
